# Endovascular Repair of Iliac Aneurysms Using the Gore Iliac Branch Endoprosthesis with Up-and-Over Technique

**DOI:** 10.3400/avd.oa.24-00114

**Published:** 2025-03-04

**Authors:** Takuya Shimizu, Miho Kamakura, Yoshihisa Murata, Kazuhiro Ota, Miki Takeda, Wakiko Hiranuma, Takayuki Matsuoka, Tadanori Minagawa, Shunsuke Kawamoto

**Affiliations:** Department of Cardiovascular Surgery, Tohoku Medical and Pharmaceutical University Graduate School of Medicine, Sendai, Miyagi, Japan

**Keywords:** abdominal aortic aneurysm, endovascular aneurysmal repair, iliac branch endoprosthesis, internal iliac artery, up-and-over technique

## Abstract

**Objectives:** The Gore iliac branch endoprosthesis (IBE) enables internal iliac artery (IIA) reconstruction, extending the indications of endovascular aneurysmal repair (EVAR); however, the up-and-over technique is challenging. This study aimed to clarify the advantages and procedural limitations of the up-and-over technique.

**Methods:** From January 2019 to October 2022, 22 patients who underwent IIA reconstruction with Gore IBE were enrolled. The patients were divided into the S and Up groups that underwent IIA reconstruction using the standard and up-and-over techniques, respectively. Aortic anatomic measurements, surgical factors, and postoperative outcomes were examined.

**Results:** No significant differences in operative time, fluoroscopy time, contrast medium use, blood loss volume, and length of postoperative hospital stay were observed between the S (12 patients) and Up (10 patients) groups. However, the distance from the lower renal artery to the reconstructed IIA origin was considerably shorter in the Up group than in the S group. During the 19-month follow-up, no adverse events were observed in the Up group.

**Conclusions:** The up-and-over technique can be a valuable option for cases where IIA reconstruction is difficult with standard procedures with Gore IBE. Therefore, understanding the procedural precautions and ensuring safety are crucial to its success.

## Introduction

Endovascular aneurysmal repair (EVAR) is a minimally invasive procedure used to treat abdominal aortic aneurysms (AAA).^1,2)^ However, approximately 20–30% of patients with AAA have iliac artery aneurysms.^3)^ In such cases, if at least one internal iliac artery (IIA) is not preserved, EVAR cannot be performed because of the risk of pelvic visceral ischemia.^4)^ To address this challenge, the Gore iliac branch endoprosthesis (IBE; W.L. Gore & Associates, Flagstaff, AZ, USA) was introduced as a device to reconstruct the IIA in EVAR.^5)^ In Japan, Gore IBE has been covered by insurance since August 1, 2017. The use of Gore IBE is expected to broaden the indications for EVAR in patients with AAA accompanied by bilateral common iliac artery (CIA) aneurysms. The standard technique involves inserting an iliac branch component (IBC) on the side where the IIA is to be reconstructed, followed by inserting an internal iliac component (IIC) from the contralateral side to complete IIA reconstruction. A bridge stent graft (SG) is inserted after the main body is in place. Mastering this technique requires specific training. Furthermore, the use of Gore IBE has anatomical limitations. It must be a minimum length of 165 mm from the low renal artery to the IIA bifurcation to be reconstructed, as the main body and IBC must be converted via a stent graft.

The up-and-over technique, in which a 12Fr sheath is guided beyond the flow divider of the bifurcated EVAR device (main body) to the contralateral side, is a procedure for IIA reconstruction in the treatment of type 1B endoleak (EL).^6)^ Using this endovascular procedure, the IBC can be inserted after the bridge stent graft is placed; therefore, IBE can be used for IIA reconstruction if the length from the inferior renal artery to the IIA bifurcation is at least 135 mm. This technique further expands the indications for Gore IBE. Despite its advantages, the up-and-over technique has not been widely adopted because it is technically demanding, and reports on its application for IIA reconstruction in initial EVAR cases are lacking.

IIA reconstruction was performed using the up-and-over technique. This study aimed to clarify the advantages and procedural limitations of the up-and-over technique.

## Materials and Methods

### Study design and participants

From January 2019 to October 2022, 22 patients who underwent IIA reconstruction with Gore IBE were enrolled. Patients deemed suitable for IIA reconstruction using the standard technique were assigned to the S group. Those with a distance of less than 165 mm from the lower renal artery to the IIA branch, or a CIA length of less than 55 mm, who were judged to have difficulty with standard IIA reconstruction, were assigned to the Up group.

We retrospectively collected clinical data from medical records, including age, sex, height, weight, body mass index (BMI), comorbidities, renal function, aneurysm diameter, site of aneurysm, and diameter of the central stationary part of the stent graft as preoperative factors. Furthermore, we collected data on surgical procedures, combined techniques, operative time, blood loss volume, fluoroscopy time, contrast medium use, and number of stent grafts used as surgical factors. Lastly, data on postoperative complications, EL, presence of reconstructed IIA patency, postoperative hospital stay, and postoperative survival were obtained as postoperative factors. The clinical data were compared between the two groups.

### Statistical analysis

Continuous variables are presented as mean ± standard deviation or as median with interquartile range. Student’s *t*-test, Mann–Whitney’s U-test, or χ^2^ test was used to compare variables between the two groups. The significance level was set at *p* < 0.05.

### Ethical considerations

The Institutional Review Board of Tohoku Medical and Pharmaceutical University Hospital approved this study (2022-2-064).

## Results

The mean age was 72.3 years in the S group and 71.7 years in the Up group, with BMI values of 24.9 ± 2.8 and 24.8 ± 2.8, respectively, showing no significant difference between the groups. The incidence of chronic obstructive pulmonary disease was higher in the S group; however, no significant differences were observed in smoking history, hypertension, diabetes mellitus, renal dysfunction, American Society of Anesthesiologists physical status, or clinical frailty scale between the groups (**Table 1**).

**Table:** Table 1 Baseline characteristics of all patients in the standard and up-and-over groups

	Standard	Up-and-over	*p*-value
N	12	10	
Age (years)	72.3 ± 4.8	71.7 ± 5.1	0.779
Male	11 (92)	10 (100)	0.328
Height (cm)	165.7 ± 6.2	162.3 ± 5.6	0.221
Weight (kg)	68.2 ± 8.1	64.2 ± 9.7	0.333
BMI	24.9 ± 2.8	24.8 ± 2.8	0.66
Smoking	12 (100)	9 (90)	0.262
COPD	10 (83)	4 (40)	0.035
Hypertension	11 (92)	9 (90)	0.892
Diabetes	5 (42)	2 (20)	0.277
CKD	2 (17)	2 (20)	0.752
CAD	6 (50)	2 (20)	0.145
CVD	2 (17)	1 (10)	0.65
Hostile abdomen	1 (8.3)	3 (30)	0.13
ASA-PS (2/3/4)	2/9/1	6/4/0	0.091
CFS (3/4/5)	7/3/2	6/2/2	0.953
Malignancy	0	2 (20)	0.104

Data are presented as mean ± standard deviation (SD), number (%), or median (IQR).

BMI: body mass index, CKD: chronic kidney disease, CAD: coronary artery disease, COPD: chronic obstructive pulmonary disease, CVD: cerebrovascular disease, ASA-PS: American Society of Anesthesiologists physical status, CFS: clinical frailty scale

**Table 2** presents the anatomical characteristics of the aneurysms in both groups. Mean aneurysm diameters were 51.6 ± 8.3 mm and 51.0 ± 8.8 mm in the S and Up groups, respectively, with no significant differences in mean aneurysm diameter, proximal neck length, and proximal neck diameter between the groups. The total iliac artery lengths were 55.8 ± 17.1 mm and 49.9 ± 18.7 mm in the S and Up groups, respectively, with no significant difference. However, the lengths from the lower renal artery to the IIA were 190 ± 16.9 mm and 167 ± 23.6 mm in the S and Up groups, respectively, which was significantly shorter in the Up group (*t*-test, *p* = 0.012).

**Table:** Table 2 Anatomical characteristics of aneurysm and comparison between the groups

	Standard	Up-and-over	*p*-value
Aneurysm diameter (mm)	51.6 ± 8.3	51.0 ± 8.8	0.881
Proximal neck length (mm)	34.2 ± 8.8	31.5 ± 10.6	0.545
Proximal neck diameter (mm)	23.7 ± 3.0	24.0 ± 3.6	0.824
Length from RA to IIA (mm)	190 ± 16.9	167 ± 23.6	0.012
Length of CIA (mm)	55.8 ± 17.1	49.9 ± 18.7	0.45

Data are presented as mean ± standard deviation (SD).

IIA: internal iliac artery, RA: renal artery, CIA: common iliac artery

**Table 3** presents the surgical factors and procedures performed simultaneously with EVAR. IIA embolization was performed in four (33%) and four (40%) patients in the S and Up groups, respectively, and IMA embolization was performed in four (33%) and two (20%) patients in the S and Up groups, respectively. Two (17%) patients in the S group underwent bilateral internal iliac reconstruction. One patient in the S group (8.3%) underwent thoracic endovascular aortic repair for a descending thoracic aortic aneurysm along with EVAR.

**Table:** Table 3 Surgical outcomes of the standard and up-and-over groups

	Standard	Up-and-over	*p*-value
Technical success	12 (100)	10 (100)	−
IMA embolization	4 (33)	2 (20)	0.746
IIA embolization	4 (33)	4 (40)	0.484
Combined with TEVAR	1 (8.3)	0	0.35
Operative time (min)	230.8 ± 66	231.5 ± 72	0.983
Intraoperative bleeding (g)	185 ± 161	335 ± 280	0.165
Fluoroscopy time (min)	54.4 ± 21.3	58.4 ± 25.7	0.709
Contrast media (mL)	95 ± 34	75 ± 32	0.182
Number of used stent-grafts	5 (5–6)	5 (5–6)	0.507

Data are presented as mean ± standard deviation (SD), number (%), or median (IQR).

IIA: internal iliac artery, TEVAR: thoracic endovascular aortic repair, IMA: inferior mesenteric artery

In comparing other surgical factors between the S and Up groups, operative time was 230.8 ± 66 vs. 231.5 ± 72 min, fluoroscopy time was 54.4 ± 21.3 vs. 58.4 ± 25.7 min, contrast medium use was 95 ± 34 vs. 75 ± 32 mL, and blood loss volume was 185 ± 161 vs. 335 ± 280 g, respectively. No significant differences were observed for any of these factors.

Five (5–6) SG were used in the S and Up groups. Two patients in each of the S and Up groups required two SGs to bridge because of suspected Type 3a EL. In the S group, an additional SG had to be implanted between the bridging SG and the IBC, and in the Up group, between the bridging SG and the main body. The success rate of the surgical procedures was 100% in both groups.

One patient in group S who underwent thoracic endovascular aortic repair and IIA embolization had postoperative complications, including access injury (external iliac artery dissection), rectal bladder injury, and lower extremity nerve and muscle injury ipsilateral to the IIA embolization. Cerebral and cardiovascular complications and renal dysfunction were not observed in either group. The median lengths of hospital stay were 5 (4.8–8.0) days and 5 (5–6) days in the S and Up groups, respectively, with no significant difference. The incidences of type 2 ELs on discharge computed tomography were 9 (75%) and 2 (20%) in the S and Up groups, respectively. No type 1A or 3 ELs occurred during the follow-up period of 46 (33–56) months and 19 (15–25) months in the S and Up groups, respectively. One patient in the S group had an IIC occlusion during the first postoperative year (**Table 4**).

**Table:** Table 4 Comparison of postoperative complications between the groups

Variables	Standard	Up-and-over	*p*-value
Postoperative death	0	0	−
Stroke	0	0	−
Cardiac events	0	0	−
Vascular access injury	1 (8.3)	0	0.35
Worsening of renal function	0	0	−
Postoperative hospital stay (days)	5 (4.8–8.0)	5 (5–6)	0.51
Follow-up period (months)	46 (33–56)	19 (15–25)	−
IIA occlusion	1 (8.3)	0	0.35
Type 1 EL	0	0	−
Type 2 EL	9 (75)	2 (20)	0.01
Type 3 EL	0	0	−
Secondary intervention	0	0	−

Data are presented as number (%) or median (IQR).

IIA: internal iliac artery, EL: endoleak

## Discussion

IIA reconstruction in EVAR is an important surgical technique to avoid critical ischemic risks, including intestinal and spinal cord ischemia, and the advent of Gore IBE has expanded the indications for EVAR treatment by avoiding bilateral IIA embolization, including AAA with bilateral CIA aneurysms. However, the anatomical limitations of Gore IBE do not always allow for IIA reconstruction with Gore IBE. The up-and-over technique is a surgical technique that can address this challenge. Tenorio et al.^7)^ evaluated the outcomes of IBE using the up-and-over technique compared to the standard technique and introduced this approach to treat type 1B EL after EVAR, using IBE to reconstruct the IIA while treating the EL. The up-and-over technique can also be used to reconstruct the IIA in cases where the distance from the renal artery to the IIA is short and IIA reconstruction is difficult with the standard technique using IBE. In the study by Tenorio et al., only 4 of 17 patients (24%) in the up-and-over group underwent primary repair, while 13 (76%) required additional treatment. In contrast, the current study compares primary aortic aneurysms with iliac aneurysms, distinguishing it from previous reports. Additionally, this study outlines the advantages, challenges, and procedural considerations of the up-and-over technique for IIA reconstruction. In other words, this study not only presents a treatment option for patients who face challenges with IIA reconstruction using standard techniques but also provides more specific technical tips for safely performing the up-and-over technique. The advantages, disadvantages, and key considerations of the procedure are detailed below.

### Advantage 1: easing anatomical limitations

**Figure 1:**
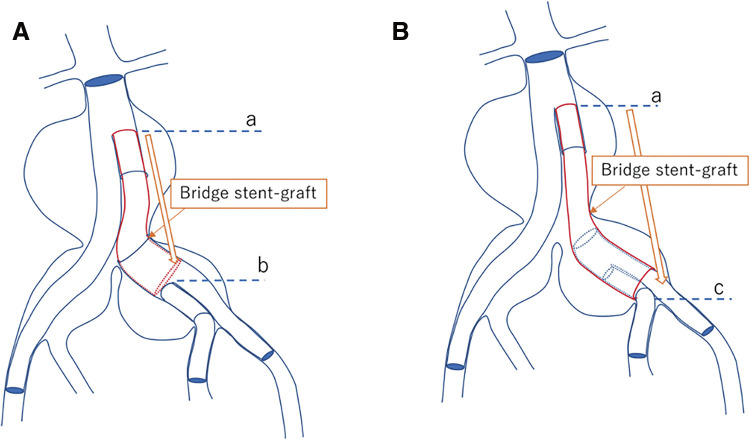
Fig. 1 (**A**) Schematic drawing of internal iliac artery reconstruction with Gore IBE stent-graft via the standard procedure. The length of the bridge stent graft is limited from the flow divider of the main body (**a**) to the iliac branch component bifurcation (**b**). However, the bridge stent graft can be chosen based on the distance from the flow divider of the main body (**a**) to the iliac bifurcation (**c**) using the up-and-over technique (**B**). IBE: iliac branch endoprosthesis

The up-and-over technique involves guiding a 12Fr sheath beyond the flow divider of a bifurcated EVAR device to the contralateral side. The greatest advantage of this technique is that it alleviates the anatomical limitation of IIA reconstruction using Gore IBE. The standard technique requires bridging the main body and IBC, meaning that the minimum distance from the lower renal artery to the IIA branch must be 165 mm (**Fig. 1A**). This is one of the anatomical limitations.

In contrast, in the up-and-over technique, the IBC is inserted after the bridge SG is placed, and the IIA is reconstructed via the IIC. Thus, the bridge SG, located outside the IBC, can be placed beyond the IBC bifurcation (**Fig. 1B**). IIA reconstruction is possible if the distal end of the bridge SG is located within the CIA. As the central diameter of the IBC is φ23 mm, a bridge SG with a peripheral diameter of φ20 mm and a minimum length of 9.5 cm would be selected. This makes IIA reconstruction possible if the length from the lower renal artery to the internal iliac branch is at least 135 mm. In this study, the distance from the lower renal artery to the internal iliac bifurcation was 167 ± 23.6 mm in the Up group, which included five (50%) patients for whom standard techniques were not indicated. However, the success rates of reconstruction and initial patency were 100%. Thus, the greatest advantage of the up-and-over technique is that it eases the anatomical limitations of the standard technique with Gore IBE, and it can contribute to expanding the indications for EVAR.

### Advantage 2: reduction of type 3a EL

IIA reconstruction using the up-and-over technique has additional advantages.

As previously stated, in the up-and-over technique, the IBC is placed after the bridge SG is implanted. As the length from the IBC bifurcation to the internal iliac gate is 2.5 cm, a bridge SG with a length of at least 2.5 cm from the internal and external iliac artery bifurcations to the periphery of the central region was selected. If a bridge SG of sufficient length is selected to reach the distal CIA, the length of the IBC and the maximum overlap (3 cm) can be secured.

Patients with strong iliac artery flexion in group S had type 3 ELs due to shallow sealing on the greater curvature side between the bridge SG and the IBC. In this case, the distance from the lower renal artery to the IIA bifurcation was 185 mm, and a 12-cm bridge SG was selected based on intraoperative measurements. If this patient had undergone the up-and-over technique, a bridge SG longer than 13.5 cm could have been chosen to ensure the IBC and overlap length. Furthermore, when SGs are implanted and overlapped at sites of high flexion, larger-diameter SGs produce a height difference on the greater and lesser curvatures. In the standard technique, a 27-mm-diameter bridge SG is inserted into the IBC. However, in the up-and-over technique, a 20-mm-diameter IBC is inserted into a 23-mm-diameter bridge SG, resulting in a smaller difference in sealing length between the greater and lesser curvatures. The up-and-over technique allows easy selection of a bridge SG, does not require fine positioning, and avoids the risk of a type 3a EL.

### Advantage 3: catheter operability

A certain degree of proficiency may be required to safely guide the 12Fr sheath to the contralateral side; however, once the 12Fr sheath can be guided, it is fixed and stabilized in an inverted U-shape in the main body, allowing stable wiring operations for IIA selection and IIC insertion. The IIC was placed in the targeted position. No type 1B EL from the IIA was observed. No IIC occlusion was observed during the follow-up period. The advantage of the up-and-over technique is that it improves catheter operation stability.

### Caution 1: possibility of sheath kinking

The 12Fr dry-seal sheath has excellent anti-kink properties; however, there is an undeniable concern regarding sheath kinking when the sheath is steeply fixed in an inverted U-shape. In this study, two patients in the Up group underwent EVAR for CIA aneurysms, and the aortic inner diameters at the site of sheath reversal were φ19 mm and φ21 mm, respectively. In both cases, the smallest diameter of the main body selected was φ23 mm; however, no trouble due to sheath kinking was observed. Sheath kinking does not occur as long as the sheath inner tube is inserted when guiding the sheath to the contralateral side and can be prevented by following basic precautions, such as not excessively pulling the pull-through guidewire through the bilateral femoral arteries after removing the sheath inner tube. However, kinking can still occur even when the aortic diameter is large; therefore, pull-through guidewire traction should be performed with caution in all cases.

### Caution 2: stent graft migration during operation

In the up-and-over technique, pull-throughs are formed from the bilateral femoral arteries via flow dividers in the main body; therefore, care should be taken to avoid migration of the main body. In this technique, the pull-through guidewire to the peripheral side of the bilateral femoral arteries should not be pulled simultaneously; however, applying tension by pulling the wire on one side to position the tip of the 12Fr sheath is crucial.

Thus, the wire should be carefully manipulated to prevent migration of the main body. Furthermore, the central landing zone of the main body before guiding the 12Fr sheath to the contralateral side must be touched and securely fixed. It is also essential to verify the position of the flow divider of the main body while performing the operation. In this study, two patients in the Up group migrated from the main body of the bridge SG. This migration occurred because the tip of the bridge SG was caught on the step between the inner and outer tubes of the sheath when the 12Fr sheath was guided to the contralateral side, and the SG moved peripherally with the sheath. After placing the bridge SG, it should touch the central landing zone of the main body, and when guiding the sheath to the contralateral side, the position of the bridge SG should be visible along with the movement of the sheath during the operation. These are the basic steps in preventing the migration of the bridge SG. Additionally, when guiding the 12Fr sheath to the contralateral side, the 16Fr sheath for IBC placement should be advanced into the main body, and the 12Fr sheath should be pulled into the 16Fr sheath. By following these precautions, migration of the bridge SG can be completely prevented.

### Caution 3: bleeding due to differences in sheath caliber

No significant difference in blood loss volume was observed between the two groups; however, more blood loss tended to occur in the Up group. In the up-and-over technique used in this study, the 18Fr sheath for insertion into the main body had to be downsized to a 12Fr sheath with a smaller diameter. Therefore, EVAR was performed using the cut-down technique owing to concerns regarding bleeding from the sheath puncture site. Certain measures, such as applying double-width sutures to the sheath puncture site, were considered necessary to control bleeding from the sheath puncture site as much as possible. Recently, percutaneous EVAR using Perclose ProGlide (Abbott, Abbott Park, IL, USA) as a hemostatic device in a pre-close technique has been performed at many medical institutions, and the hospital stay has been shortened owing to the reduced risk of wound infection and lymphatic leakage. When a sheath is downsized, bleeding from the sheath puncture site can be prevented by pulling the axonemal thread of Perclose ProGlide, which can be applied to EVAR using the up-and-over technique to reduce blood loss.^8)^

### Caution 4: length of delivery system

In the up-and-over technique, the contralateral IIA is reconstructed using a flow divider of the main body. Therefore, the reach to the contralateral IIA is longer than that in the standard technique, and the IIC may not reach the target implantation site. In this study, all patients in the Up group were men, and their height, weight, and BMI were averaged for the Japanese population. None of the patients had difficulty in IIA reconstruction because the IIC did not reach the target implantation site. In patients with a long distance from the contralateral IIA owing to the meandering of the iliac artery, a 12Fr sheath can be sufficiently guided to the contralateral side by straightening the iliac artery using a stiff guidewire. If the patient is Japanese and has a standard physique, the IIA can be reconstructed using the up-and-over technique. However, it can be reliably determined during surgery whether the IIC can reach the targeted implantation site by the 12Fr sheath reaching the contralateral IIA. Because the effective length of the IIC delivery catheter is 63 cm, and the total length of the 12Fr sheath is 50 cm, the tip of the IIC catheter can be advanced 13 cm from the tip of the 12Fr sheath. The length of the IIC SG is 7 cm; therefore, if the tip of the 12Fr sheath can reach at least 6 cm on the central side of the long marker at the entrance of the IIA side of the IBC bifurcation, it can be computationally determined that the IIC SG can be placed. Because the 3 cm central side of the long marker is the central edge of the IBC, it can be easily judged during surgery using the central edge of the IBC or the central edge marker as a guide. If the tip of the 12Fr sheath can be advanced to the central edge of the IBC, the IIC can be placed with room spared. If the IIC delivery catheter is deemed inaccessible, the Gore Viabahn VBX balloon expandable SG with an effective length of 80 cm can be used for IIA reconstruction. If VBX SG are used, no significant differences are observed in the IIA patency rate, EL, or prognosis; VBX is considered a useful alternative device to IIC.^9)^ However, the use of VBX for IIA reconstruction is not yet covered by insurance. It is recommended that the VBX be prepared as a backup if the IIC catheter does not reach the targeted implantation site. Despite these limitations, we were able to complete IIA reconstruction in this study without increasing the operative time, fluoroscopy time, or contrast media usage. Therefore, if the surgeon is skilled in the EVAR technique, the up-and-over technique can be a valuable option for cases where IIA reconstruction is difficult with standard procedures and is deemed important to avoid serious complications such as intestinal or spinal cord ischemia.

### Study limitations

The current study has some limitations that should be noted. This was a retrospective study with a short follow-up period. Selection bias is possibly at play in cases in which the up-and-over technique was used; for instance, cases that were considered technically easier to perform might have been selected. Additionally, cases where the distance from the renal artery to the IIA was longer, allowing IIA reconstruction with the standard technique, may have made IIA reconstruction difficult using the up-and-over technique.

Nevertheless, because IIA is difficult to reconstruct with IBE using the standard technique in cases with short distance from renal artery to IIA, it is not possible to compare techniques, and prospective randomization cannot therefore be performed. Rather, the up-and-over technique is remarkable in that it allows for IIA reconstruction with Gore IBE without increasing surgical complications among patients in whom reconstruction cannot be performed using the standard technique. While no significant difference in the reconstructed IIA patency rate was observed between the two groups during the follow-up period, further long-term follow-up may reveal a significant difference in the patency rate due to the difference in bridge stent graft diameters.

## Conclusion

The up-and-over technique can be a valuable option for cases where IIA reconstruction is difficult with standard procedures with Gore IBE. The short-term results confirm that the up-and-over technique is a useful procedure that can overcome anatomical limitations. Nonetheless, understanding the advantages and disadvantages of this endovascular procedure is important to ensure its safety, and further long-term follow-up is necessary to validate the results.

## Declarations

### Acknowledgments

The authors thank Editage (www.editage.com) for English language editing.

### Disclosure statement

All authors declare no conflict of interest.

### Author contributions

Study conception: TS and SK

Data collection: TS

Analysis: TS and SK

Investigation: TS

Manuscript preparation: TS, MK, and SK

Funding acquisition: None

Critical review and revision: all authors

Final approval of the article: all authors

Accountability for all aspects of the work: all authors.
